# Karyological studies in ten species of *Citrus*(Linnaeus, 1753) (Rutaceae) of North-East India

**DOI:** 10.3897/CompCytogen.v5i4.1796

**Published:** 2011-11-09

**Authors:** Marlykynti Hynniewta, Surendra Kumar Malik, Satyawada Rama Rao

**Affiliations:** 1 Plant Biotechnology Laboratory, Department of Biotechnology and Bioinformatics, North-Eastern Hill University, Shillong (Meghalaya) India; 2 Tissue Culture & cryopreservation Unit, National Bureau of Plant Genetic Resource, New Delhi

**Keywords:** *Citrus*, karyotype, genetic variability, asymmetry index

## Abstract

Abstract

Ten *Citrus* (Linnaeus, 1753) species of North-East India have been karyo-morphologically analysed. All studied species had 2n=18 chromosomes without any evidence of numerical variation. All the chromosomes were found to be of metacentric and sub-metacentric in all the species; the morphology of the chromosomes showing size difference only. Symmetrical karyotype which does not have much difference in the ratio of longest to shortest chromosome in all the species was observed. Three species, *Citrus grandis* (Osbeck, 1757), *Citrus reticulata* (Blanco, 1837) and *Citrus medica* (Linnaeus, 1753) are identified as true basic species from asymmetry studies of karyotypes as they reflect on the primitive nature of their genomes. *Citrus indica* (Tanaka, 1937)occupies a special taxonomic position within the genus *Citrus* as a progenitor for other cultivated species.

## Introduction

The genus *Citrus* is economically very important and is known for its juice and pulp throughout the world. The genus belongs to the family Rutaceae that includes 162 species (Tanaka 1977) and is grown in tropical and subtropical areas of the world. *Citrus* is the third most important fruit crop of India with an estimated production of 4.2 million tons from an area of 0.48 m ha (Bathla et al. 2001). Mandarin (*Citrus reticulata* Blanco, 1837), sweet orange (*Citrus sinensis* Osbeck, 1757), acid lime (*Citrus aurantifolia* Swingle, 1913) and lemon (*Citrus limon* Osbeck, 1765) are the major cultivated species of the country. Other species that are cultivated to a lesser extent include seedless lime (*Citrus latifolia* Tanaka, 1937), pummelo (*Citrus grandis* Osbeck, 1757), grapefruit (*Citrus paradisi* Macfadyen, 1930) and belladikithuli (*Citrus maderaspatana* Tanaka, 1937). In India, there are 30 species of *Citrus* (Singh and Chadha 1993) of which at least nine species are available throughout India, while 17 species are confined to North-Eastern India. It is also reported that nine species are found in the southern region of India, six species in the north-western India while a single species is observed in central region of the country (Singh and Chadha 1993). The north-east region of India is known for its rich diversity in *Citrus* germplasm, reflected in 17 species, 52 cultivars and 7 probable natural hybrids which are found in the region (Bhattacharya and Dutta 1956). A recent study on genetic resources of *Citrus* from north-eastern India indicated an increase in the number of species up to 23 besides one subspecies and 68 varieties (Sharma et al. 2004). *Citrus* plants growing in deep forests undisturbed by biotic factors have also been reported from the region, thus bestowing this area with a special status of “treasure house” of *Citrus* germplasm and also highlighted the lack of our knowledge about the same (Sharma et al. 2004).

The south-east Asia, Australia and the intervening island-areas between Australasia and Central Africa and the north-eastern region of India along with neighbouring China (Mc Phee 1967, Swingle and Reece 1967) are thought to be important centres of origin of *Citrus* and related genera. Many *Citrus* species are believed to be endemic to the region. Seven Indian *Citrus* species fall under the category of endangered species which include *Citrus indica* Tanaka, 1937, *Citrus macroptera* Montrouzier, 1960, *Citrus latipes* Tanaka, 1937, *Citrus assamensis* Dutta et Bhattacharya, 1956, *Citrus ichangensis* Swingle, 1913, *Citrus megaloxycarpa* Lushington, 1910 and *Citrus rugulosa* Tanaka, 1937(Malik et al. 2006). Two species, *Citrus indica* and *Citrus macroptera*, need special and immediate attention for conservation due to their endemism and high degree of threat perception.

South and western hills of Meghalaya in the North-East are reported to have maximum diversity for *Citrus reticulata*, *Citrus grandis*, *Citrus limon* and *Citrus aurantifolia*. These are extensively cultivated for their taste, good pulp and have very high market demand. *Citrus indica* is supposed to be the most primitive species and perhaps the progenitor of cultivated *Citrus* (Malik et al. 2006) and is locally known as Memang Narang. It is a rare species which is confined to the Tura ranges of West Garo Hills (Upadhyay and Sundriyal 1998). *Citrus macroptera* is reported to grow in the Khasi and Garo Hills of Meghalaya, North Cachar, Karimganj and Karbi-Anglong districts of Assam and the states of Mizoram, Tripura and Manipur (Bhattacharya and Dutta 1956, Sharma et al. 2004). *Citrus megaloxycarpa* locally known as ‘Sishupal’ is a rare species, confined to the Jampui Hill regions of Mizoram and *Citrus latipes* shows maximum occurrence in West Khasi Hills of Meghalaya.

The relationship between the species within the genus *Citrus* has been made complicated due to combination of factors such as wide cross compatibility, repeated cross pollination and apomixis. Wide hybridization in *Citrus* affects karyotype stability (Khan 2007). Hybridization has probably played an important role in the evolution of most *Citrus* species. Scora (1975) and Barrett and Rhodes (1976) suggested that there are only three basic species of *Citrus*, that are considered true ones within subgenus *Citrus* while other species within this subgenus are hybrids derived from the three true species or by intercrossing with species of subgenus *Papeda* (Swingle, 1943) or other closely related genera. Wild relatives of cultivated *Citrus* species can be a major source of genetic variation for utilization in breeding programs aimed at crop improvement through transfer of disease resistance or other desirable agronomic traits.

The cytogenetical characterization of *Citrus* accession could help in the identification of a particular genomic variant, or for the detection of true hybrids in breeding program, as well as for studies of karyotypes evolution of the group (Guerra et al. 1997). Despite the great genetic diversity and economic significance attached to several species of *Citrus*, attempts to understand the genetic basis of variation is not forth coming. The available information is scant and fragmented. A quick perusal of the published literature indicates different chromosome number reports in several species such as 2n=18 or 2n=27 in *Citrus aurantifolia* (Longley 1925; Krug and Bacchi 1943) and 2n=18, 27, 36 in *Citrus limonia* Osbeck, 1757 (Frost 1925a, b) are case examples. Therefore there is an urgent need to undertake comprehensive cytogenetical approaches to define the existing genetic variation at inter- and intra-specific levels in the genus *Citrus*. The present investigations are an attempt to conduct karyomorphological studies on 10 species of *Citrus* from North-East India.

## Material and methods

The plant material used in the present investigation was collected from various region of North-East India and the vouchers specimens have been submitted to National Herbarium of Crop Plants, National Bureau of plant Genetics Resources, New Delhi (Table 1). The plants were grown in green house of Plant Biotechnology Laboratory, Department of Biotechnology and Bioinformatics of North-Eastern Hill University, Shillong. For each species, wherever possible, a minimum of five individuals and more than one population were analyzed. For obtaining actively growing root tips, plants were raised in earthen pots and the root tips of about (0.5–1.0 cm) long were excised. All the root tips were pre-treated with 8-hydroxyquinoline (0.002M) for three hours at room temperature, fixed in ethanol-acetic acid (v/v, 3:1) and subsequently stored at 4^o^C until required. For slide preparation, the root tips were washed twice in distilled water, hydrolysed in 5N HCl for 20 min at room temperature. The hydrolysed root tips were washed in distilled water and stained in Feulgen stain for 45 min. The root tips were subsequently squashed in 1% acetocarmine. The micro-photographs were taken using Jenoptik CCD camera (Germany) attached to labomed LX 400 brightfield microscope. At least five clear preparations of chromosome complements of each species were analyzed for the karyotypes. Idiograms were prepared from photo-micrographs by cutting out individual chromosomes, arranging them in descending order of their length and matching on the basis of morphology. The standard method of chromosome classification (Levan et al. 1964) of metacentric (V), submetacentric (L), subtelocentric (J) and telocentric (I) based on the arm ratio of 1:1, >1:1<1:3, >1:3<1:0 and 1:0 respectively, was used for comparison. The degree of symmetry was estimated as per the scheme proposed by Paszko (2006).

**Table 1.** *Citrus* species used in the present investigation.

**Sl. No.**	**Species**	**Common Name**	**Collection No.**	**Source**
**Subgenus Citrus**				
1	*Citrus reticulata*	Khasi Mandrin	CR-9	Pynursla
2	*Citrus jambhiri*	Rough lemon	CJ-6	Wahkhen
3	*Citrus sinensis*	Sweet orange	CS-2	Shillong
4	*Citrus limon*	Assam Lemon	MD/33	Mizoram
5	*Citrus grandis*	Pummelo	CG-7	Ri Bhoi
6	*Citrus limetta*	Sweet limes	CLe-1	Shillong
7	*Citrus indica*	Indian wild orange	SO1	Nokrek, Garo hills
8	*Citrus medica*	Citron	CMi-2	Wahkhen
**Subgenus Papeda**				
9	*Citrus macroptera*	Melanesian Papeda	CMa-1	Cherrapunjee
10	*Citrus latipes*	Khasi Papeda	Clt-2	Upper Shillong

## Results

**Figure 1.**
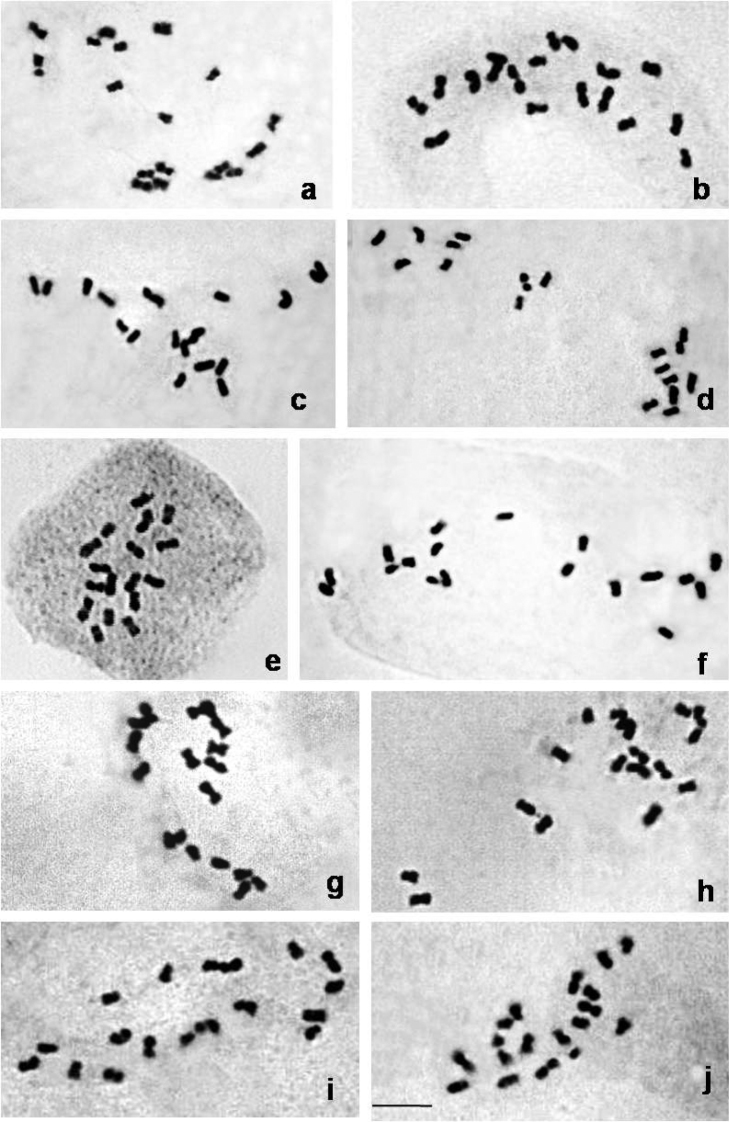
Mitotic complements of 10 *Citrus* species (2n=2x=18). **a** *Citrus macroptera*, **b** *Citrusgrandis*, **c** *Citrus medica*, **d** *Citrus reticulata*, **e** *Citrus sinensis*, **f** *Citrus jambhiri*, **g** *Citrus latipes*, **h** *Citrus indica*, **i** *Citrus limon*, **j** *Citrus limetta*. Bar = 5µm.

**Figure 2.**
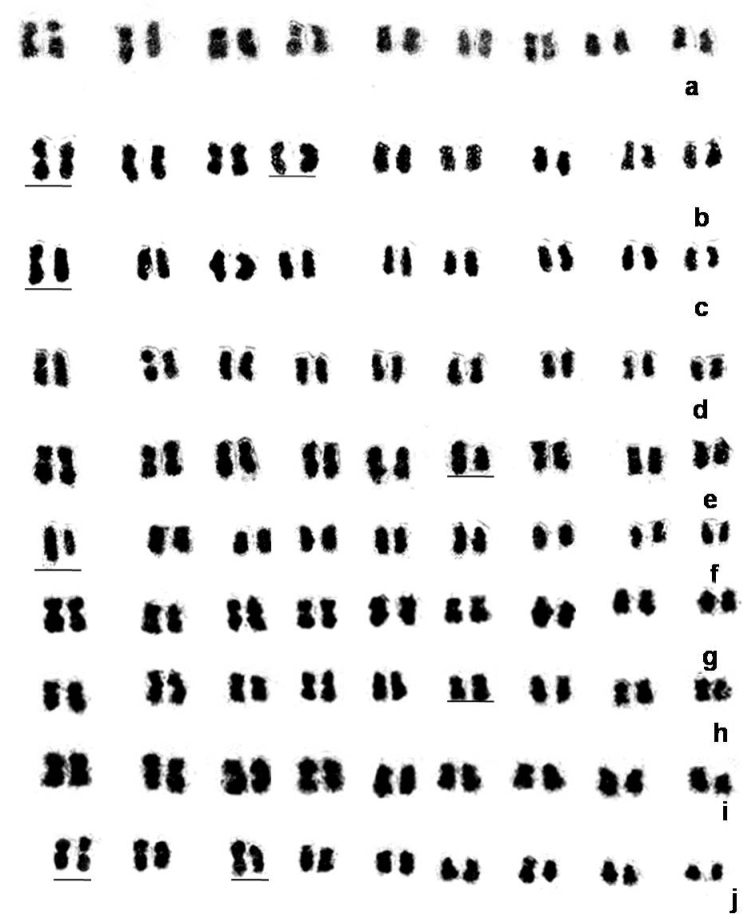
Karyograms of 10 *Citrus* species. **a** *Citrus macroptera*, **b** *Citrus grandis*, **c** *Citrus medica*, **d** *Citrus reticulata*, **e** *Citrus sinensis*, **f** *Citrus jambhiri*, **g** *Citrus latipes*, **h** *Citrus indica*, **i** *Citrus limon*, **j** *Citrus limetta*. Bar represent heteromorphic pairs.

The data related to chromosome complements/karyotypes have been presented in Table 2 and illustrated in Fig. 1 and 2 and it is amply clear that among the 10 species of *Citrus* presently studied, two species namely *Citrus jambhiri* Lushington, 1910 and *Citrus limon* Linnaeus, 1753 were characteristic in having exclusively sub-metacentric chromosomes in the chromosomes complements. On the other hand the remaining 8 species namely *Citrus macroptera*, *Citrus grandis*, *Citrus medica* Linnaeus, 1753, *Citrus reticulata*, *Citrus sinensis*, *Citrus latipes*, *Citrus indica* and *Citrus limetta* Linnaeus, 1753 had at least one pair of metacentric chromosome among the chromosome complements. It was more intriguing to record that two metacentric pairs were observed in *Citrus reticulata* and *Citrus latipes* as metacentrics while one pair of metacentric were recorded in remaining 6 species. Further the position of the meta-centrics varied in different species of *Citrus* presently studied ranging from 2^nd^ pair (in *Citrus grandis* and *Citrus latipes*), 3^rd^ pair (in *Citrusreticulata*), 4^th^ pair (in *Citrus macroptera* and *Citrus indica*), 5^th^ pair (in *Citrusreticulata*, *Citrus latpipes* and *Citrus limetta*), 7^th^ pair (in *Citrus sinensis*) and 8^th^ pair (in *Citrus medica*). Thus, the 6^th^ and 9^th^ pairs in all the species have been found to be invariably sub-metacentric.

**Table 2.** Karyotype formulae and characteristics in 10 species of *Citrus*. AI- asymmetry index; SC - the shortest chromosome length; LC - the longest chromosome length; CL - mean length of chromosome; CI - mean centromeric index; SD - standard deviation; CV_CL_- component expressing the relative variation in chromosome length; CV_CI _- component expressing the relative variation in centromeric index.

**Species**	**Collection No**	**2n**	**Number of second-dary constriction**	**Range SC-LC (μm)**	**Ratio LC/SC**	**CL (μm) Mean (±SD)**	**CI Mean (±SD)**	**C_VC_L**	**C_VC_I**	**AI**	Karyotype formula*
*Citrus macroptera*	Cma-1	18	-	5.01–10.52	2.09	7.44 (±1.9)	40.99 (±5.4)	25.53	13.17	3.36	16L+2V
*Citrusgrandis*	CG-7	18	2	4.03–11.12	2.75	7.83 (±2.04)	43.93 (±3.2)	26.05	7.28	1.89	16L+2V
*Citrusmedica*	Cmi-1	18	-	4.51–12.02	2.66	6.88 (±1.84)	42.73 (±3.0)	26.74	7.02	1.87	16L+2V
*Citrusreticulata*	CR-9	18	-	4.01–9.03	2.25	6.51 (±1.5)	43.07 (±4.6)	23.04	10.68	2.46	14L+4V
*Citrus sinensis*	CS-2	18	-	4.03–9.51	2.35	6.71 (±1.34)	43.88 (±6.7)	19.97	15.26	3.04	16L+2V
*Citrus jambhiri*	CJ-6	18	-	4.04–9.02	2.23	5.51 (±1.2)	38.72 (±6.7)	21.77	17.3	3.76	18L
*Citrus latipes*	CLt-1	18	-	4.02–10.11	2.51	7.08 (±1.76)	39.97 (±6.9)	24.85	17.26	4.28	14L+4V
*Citrus indica*	SO1	18	-	4.01–8.14	2.02	5.81 (±1.28)	43.1 (±3.8)	22.03	8.81	1.94	16L+2V
*Citrus limon*	MD/33	18	2	4.03–9.10	2.25	6.38 (±1.39)	42.16 (±5)	21.78	11.85	2.58	18L
*Citrus limetta*	Cle-1	18	-	3.51–9.01	2.56	6.18 (±1.77)	42.48 (±4.8)	28.64	11.29	3.23	16L+2V

* As per the method of Levan et al 1964

Sub-telocentric and telocentric chromosomes which are presumed to significantly influence the symmetry of the karyotype were alltogether absent in any of the species presently studied. From the details of karyotypic formula derived for various species of *Citrus*, three patterns of karyotype formulae, 18L, 16L+2V and 14L+4V, were recorded. The ratio of longest to shortest chromosomes was recorded as highest in *Citrus grandis* and the lowest in *Citrus indica*.

Partial homology among the somatic chromosomes is often expressed in the form of heteromorphism and heteromorphic pairs in karyotypes. The present observation of 10 different species of *Citrus* had shown interspecific diversity with regards to presence or absence of heteromorphic pair in the chromosome complements. *Citrus macroptera*, *Citrus reticulata*, *Citrus limon* and *Citrus latipes* were characteristic in lacking any heteromorphic pair, while *Citrus grandis*, *Citrus medica* and *Citrus limetta* are unique in having two pairs of heteromorphic chromosomes in their respective complements. One pair of heteromorphic chromosomes was characteristic in *Citrus sinensis*, *Citrus jambhiri* and *Citrus indica*.

Due to technical problems nucleolar chromosome could not be clearly scored in any of the species presently studied, although there were some indications to suggest that the second pair in *Citrus grandis* and third pair in *Citrus limon* are probably nucleolar in nature by revealing the secondary constriction.

The asymmetry index (AI) value which has been derived from the data related to Chromosome length (CL) and Centromeric index (along with the co-efficient of variation) has resolved the ten species of *Citrus* presently investigated into two groups, one with low value of asymmetry index indicating high karyotype symmetry corresponding to *Citrus medica* (1.87), *Citrus grandis* (1.89), *Citrus indica* (1.94), *Citrus reticulata* (2.46). The other group with high asymmetry index indicate low karyotype symmetry corresponding to *Citrus sinensis* (3.04), *Citrus limetta* (3.23), *Citrus macroptera* (3.36), *Citrus jambhiri* (3.76) and *Citrus latipes* (4.28). *Citrus limon* reported to be an intermediate species had an asymmetry index value of 2.58 indicating its link between the above two groups.

## Discussion

From the perusal of published literature it can be seen that the somatic chromosome number in the genus *Citrus* is diverse ranging from 2n=18, 27, 36, 54, etc. (Bacchi 1940; Krug 1943; Krug and Bacchi 1943; Lapin 1937) in various species. It can be seen from the above published data, that the relationship is indicative of a probable polyploid series with a basic number of x=9. In the present investigation all the somatic cells analysed in 10 different species had 2n=18. However in one specimen of *Citrus reticulata* 2n=36 was recorded. Thus the present studies involving 10 representative species did conform the somatic chromosome number as 2n=18 only without any exception.

Thus the present data as reflected from Fig. 1 and 2, combined with chromosome counts available from the literature confirms that the genus *Citrus* is apparently monobasic in nature and x=9 is the most acceptable number. Such observation received an ample support from reports of Krug (1943), Tanaka (1930), Yamamoto et al. (2007), and Barros e Silva et al. (2010). The sporadic occurrence of 2n=36 in a few cells of *Citrus reticulata* is another indication for x=9 as the true basic number of the genus *Citrus*. The basic chromosome number of *Citrus* (Rutaceae) and other related genera of the subfamily Aurantioideae has been reported as x=9 (Frost 1925). The majority of the wild and cultivated forms of *Citrus* are identified as diploids, i.e. 2n=2x=18 (Krug 1943). However polyploids are known to exist, which arise either spontaneously or following certain cross combination. For example there have been reports of naturally occurring tetraploids from inter-specific crosses between tetraploid and diploid taxa (Oiyama et al. 1991) and induced polyploids by colchicine (Barret 1974, Oiyama and Okudai 1986). Heteroploid crosses involving tetraploid (4x) and diploid (2x) species resulted in spontaneous production of a triploid ‘Tahiti lime’ (Krug and Bacchi 1943; Oiyama et al. 1991, 1980). Luss (1935) was the first to report about a hypertriploid (3x +1=28). Similar observations of hypertriploid were also reported by Lapin (1937), Krug and Bacchi (1943) who have recorded the occurrence of aneuploid from the progeny of various crosses among diploid species. Inter-specific hybridization, ploidy level and the mono/polyembryonic nature of the *Citrus* variety may also contribute to the frequency of polyploid progenies (Cameron and Soost 1969; Wakana et al. 1981).

In the present studies on 10 different *Citrus* species, the chromosome complements were all resolved into either metacentric or sub-metacentric chromosomes only. From the details of karyotypic formulae derived for these species of *Citrus*, three patterns of karyotype formulae, 18L, 16L+2V and 14L+4V, were recorded and there was complete absence of sub-telocentric and telocentric chromosomes which is indicative of the stability of the genome and of the absence of structural alteration of the chromosomes in the genus *Citrus*. Therefore, it is presumed that speciation in the genus *Citrus* could have been influenced by gene mutations which have no effect in the overall structure of chromosomes.

Swingle and Reece (1967), opined that the genus *Citrus* has only three ‘basic’ true species viz. Citron (*Citrus medica* L.), Mandarin *(Citrus reticulata* Blanco), and Pummelo (*Citrus grandis* Osbeck), while the rest of the species are hybrid derivatives of any one of the true species and species belonging to sub genus *Papeda* (Barrett and Rhodes 1976; Federici et al. 1998; Nicolosi et al. 2000; Scora 1975). However the high resolution of karyotypes as observed in the present mitotic preparations does not distinguish between basic true species and derived ones. There was no grouping of chromosomes for distinguishing the karyotypes on the basis of the hybrid nature of species as reported. However, the staining methods used traditionally with aceto-carmine, aceto-orcein or Feulgen’s solution were less informative to reveal detailed structure under the usual optical microscope because the mitotic chromosomes are very small (1.0–4.0 μm) and most of them are similar in morphology (Krug 1943). Therefore, to establish the hybrid nature of some of the species can only be determine by using more sensitive technique like *in situ* hybridization and the study of banding patterns of the chromosomes.

From the karyological data presented in Table 2 it can be observed that the asymmetry index of different species of *Citrus* presently investigated had shown significant variation. *Citrus medica*, *Citrus grandis* and *Citrus reticulata* which are considered as true basic species (Swingle and Reece 1967) are characteristic in having low asymmetry index of 1.87, 1.89 and 2.46 respectively. On the other hand 6 species had higher asymmetry index while *Citrus indica* had an intermediate value. The lower asymmetry indexes of the 3 species recorded suggest an ancestral genome which makes them as true basic species. The higher asymmetry index value recorded in 6 species is indicative of the fact that their genomes are relatively advanced and are in a process of reorganisation through chromosome structural alterations. *Citrus indica* with its intermediate value of asymmetry index may be regarded as one of the progenitor species of cultivated *Citrus* (Malik et al. 2006) and has a special position in the genus.
